# Frequency of MTHFR G1793A polymorphism in individuals with early coronary artery disease: cross-sectional study

**DOI:** 10.1590/1516-3180.2013.1315500

**Published:** 2013-10-01

**Authors:** Antonio Ivo Moritz, Joel Rolim de Moura, Darlene Camati Persuhn

**Affiliations:** I MD. Physician, Ultratito Clinic, Florianópolis, Santa Catarina, Brazil.; II MD. Physician, Municipal Healthcare, Itajaí, Santa Catarina, Brazil.; III PhD. Biochemist and Assistant Professor I, Department of Molecular Biology, Universidade Federal da Paraíba, João Pessoa, Paraíba, Brazil.

**Keywords:** Atherosclerosis, Homocysteine, Cardiovascular diseases, Polymorphism, restriction fragment length, Hyperhomocysteinemia, Aterosclerose, Homocisteína, Doenças cardiovasculares, Polimorfismo de fragmento de restrição, Hiper-homocisteinemia

## Abstract

**CONTEXT AND OBJECTIVE:**

Atherosclerotic disease is the leading cause of death in Brazil. It is a complex disease and its prevention involves identification and control of risk factors. Moderately increased plasma homocysteine concentration (hyperhomocysteinemia) has been considered to be a risk factor for several vascular diseases. Mutations in the methylenetetrahydrofolate reductase (MTHFR) enzyme, which is involved in homocysteine metabolism, have been investigated as potential vascular disease risk factors. G1793A polymorphism was described in 2002 and there are few studies analyzing its involvement in diseases. The objective of this study was to investigate the prevalence of G1793A polymorphism in subjects with early coronary artery disease (CAD).

**DESIGN AND SETTING:**

Cross-sectional study with control group conducted at a private cardiology clinic and a molecular biology laboratory (Universidade do Vale do Itajaí).

**METHODS:**

We studied 74 early-onset CAD+ patients and 40 CAD- individuals with normal angiography results. DNA was extracted from blood samples. Molecular data were obtained via PCR/RFLP and agarose gel electrophoresis.

**RESULTS:**

The occurrence of G1793A heterozygotes was similar in the control (5%) and test (6.25%) groups, thus showing that in the population studied there was no correlation between the marker and occurrences of early CAD. There was also no association between the polymorphism and the risk factors for atherosclerosis.

**CONCLUSIONS:**

The frequency of the 1793A allele in the test group (3.4%) was similar to what was found in the control individuals (2.5%). There was no correlation between G1793A polymorphism and occurrences of early CAD in this population.

## Abstract (pt)

**CONTEXTO E OBJETIVO:**

A doença aterosclerótica é a principal causa de morte no Brasil. Trata-se de doença multifatorial e sua prevenção passa pela identificação e controle dos fatores de risco. A concentração plasmática moderadamente aumentada de homocisteína (hiperhomocisteinemia) tem sido considerada importante fator de risco para várias doenças vasculares. Mutações na enzima metilenotetrahidrofolato redutase (MTHFR), envolvida no metabolismo de homocisteína, têm sido investigadas como fatores de risco para doenças vasculares. O polimorfismo G1793A foi descrito em 2002 e existem poucos estudos sobre sua implicação em doenças. O objetivo do presente trabalho foi verificar a prevalência do polimorfismo MTHFR G1793A em indivíduos portadores de doença arteriocoronariana (DAC) precoce.

**TIPO DE ESTUDO E LOCAL:**

Estudo transversal com grupo controle realizado em Clínica Cardiológica Particular e Laboratório de Biologia Molecular (Universidade do Vale do Itajaí).

**MÉTODOS:**

Foram estudados 74 pacientes DAC+ precoce e 40 DAC- com resultado angiográfico normal. O DNA foi extraído de amostras de sangue. Dados moleculares foram obtidos através de PCR/RFLP e eletroforese em gel de agarose.

**RESULTADOS:**

A ocorrência de heterozigotos G1793A foi similar em ambos os grupos controle (5%) e teste (6,25%), mostrando que na população estudada não existiu correlação entre o marcador e a ocorrência de DAC precoce. Não houve associação entre o polimorfismo e os fatores de risco para aterosclerose.

**CONCLUSÕES:**

A frequência do alelo 1793A no grupo teste (3,4%) foi parecida com a encontrada nos indivíduos do controle (2,5%). Não houve correlação entre o polimorfismo G1793A e a ocorrência de DAC precoce na população estudada.

## INTRODUCTION

Genetic polymorphism of methylenetetrahydrofolate reductase (MTHFR) has been the subject of increasing attention as a potential genetic marker associated with atherosclerosis.^1-5^ The human 5,10-MTHFR gene is located at the end of the short arm of chromosome 1 (1p36.3), and the total length of the gene cDNA (complementary DNA) is 2.2 kb. MTHFR plays a crucial role in the metabolism of folates and irreversibly converts 5,10-methylenetetrahydrofolate to 5-methyltetrahydrofolate. 5-methyltetrahydrofolate is the predominant circulatory form of folates and donates a methyl group for remethylation of homocysteine to methionine. Consecutively, methionine is metabolized to yield S-adenosylmethionine (SAM), the main methyl donor for important methylation reactions that are required for DNA repair. Impaired MTHFR activity may lead to homocysteine accumulation in plasma, and this condition may contribute towards progressive atherosclerosis through several mechanisms, including arterial endothelial function impairment, oxidative stress induction and promotion of inflammation and thrombosis.^6-9^

Two common polymorphisms of the MTHFR gene have been detected: C677T and A1298C, which both result in some impairment of enzymatic activity. The 677C>T (Ala222Val) polymorphism occurs in exon 4 and results in alanine-to-valine substitution at codon 222. The 1298A>C (Glu429Ala) polymorphism occurs in exon 7 and results in glutamate-to-alanine substitution at codon 429. There is no consensus regarding the involvement of these polymorphisms as candidates for increasing the risk of vascular disease, although there is a vast body of literature on the subject._^1-5^_ ^,^ _^10-13^_

A third and novel polymorphic site of the MTHFR gene in exon 11 has been reported by Rady et al.^14^ The 1793G>A polymorphism results an arginine-to-glutamine change at codon 594, and no reports about its involvement in MTHFR activity have been published. The implications of G1793A as a genetic risk for non-syndromic cleft lip and/or palate,^15^ prostate cancer^16^ and cardiovascular risk in end-stagerenal disease^17^ have been reported.

### OBJECTIVE

The objective of this study was to investigate whether there is any difference in allele prevalence in MTHFR G1793A polymorphism between subjects with early CAD and subjects without lesions, as evaluated by means of coronary catheterization.

### METHODS

This cross-sectional study was conducted in a private cardiology clinic in Florianópolis, state of Santa Catarina, Brazil, and the Molecular Biology Laboratory of Universidade do Vale do Itajaí. The study protocol was approved by the university's Ethics Committee (protocol 374/2003), and all subjects gave their written informed consent before participation 

This study was conducted between August 2003 and December 2003. All patients under the age of 55 years (men) and 65 (women) who underwent coronary angiography between the years 2002 and 2003 were invited to participate. Patients were indicated to join the group test consequent to angiography results suggestive of myocardial ischemia. We excluded patients who had not undergone laboratory tests in the same week as their angiography examination. The angiographic and biochemical analyses were both performed in the same clinic. The inclusion criteria were met by 208 patients; 150 were CAD + and 58 were CAD-. The study population comprised 114 individuals who decided to participate: 74 CAD+ and 40 CAD(8.5%) sampling error; confidence level 95%).

The allocation to the groups depended on the angiography result: patients presenting stenosis in one or more coronary arteries were classified as CAD+. CAD- was defined as absence of stenosis in any coronary artery. The degrees of stenosis were determined visually by an observer. The atherosclerotic lesions in the CAD+ group were classified as mild when the obstruction was 30% (n = 6; 8.1%), moderate when the degree of arterial impairment was between 31 and 70% (n = 20; 27.4%) and serious when stenosis affected 70% or more (n = 48; 64.86%). This stratification was ignored for the analysis on the molecular results and risk factors.

### Leukocyte DNA extraction

Peripheral blood (5 ml) was collected from the subjects in a tube containing EDTA. The extraction of genomic DNA from leukocytes was performed in accordance with the method described by Miller et al.^18^

### MTHFR G1798A genotyping

The presence of the G1798A mutation was determined by amplifying a fragment of exon 11 of the gene encoding the enzyme MTHFR, by means of the polymerase chain reaction (PCR). The primers used were: 5'CTCTGTGTGTGTGTGCATGTGTGCG3' and 5'GGGACAGGAGTGGCTCCAACGCAGG3'. The parameters used in the reaction were: one initial step of two minutes at 94 °C, forty cycles of one minute of denaturation at 94 °C, one minute of annealing at 66 °C and one minute of extension at 72 °C. The reaction had one extra step of extension for 10 minutes, at 72 °C. This process generated a generated a fragment of 310 base pairs.

The amplified fragment was digested using the restriction enzyme *Bsrb*I, in accordance with the supplier's instructions, followed by electrophoresis on 1.5% agarose gel (Gibco, Brazil), stained with ethidium bromide for viewing through an ultraviolet transilluminator (Ultra Lum).

Digestion of the 310 base-pair fragment using the enzyme *Bsrb*I resulted in two bands of 233 and 77 base pairs in genotype 1793GG, while heterozygous 1793GA appeared in three bands: 310, 233 and 77 base pairs. In the presence of mutant genotype 1793AA, only the band of 310 base pairs was viewed.^14^

### Data analysis

The data were tabulated in Microsoft Excel software and the two groups (presence and absence of early CAD) were compared with regard to their biochemical profile, presence/absence of risk factors and presence of the G1793A polymorphism. The allele frequency was calculated for both groups. The Hardy-Weinberg equilibrium was calculated by determining the frequency of genotypes expected from the allele frequency determined in the study population. To compare the genotypic groups, we used the test of difference between two proportions, with a tabulated Z value for a risk of error of 5%, thus giving a value of 1.96. In quantitative data, the statistical test was Mann-Whitney, with a significance level of P < 0.05.

### RESULTS

### Demographic and clinical data

The study was conducted on 74 CAD+ individuals and 40 CAD- individuals (control group). The average ages were 52 ± 6 and 51 ± 6 in the CAD+ group (38 males and 36 females) and control group (9 males, 31 females), respectively. There was no statistical difference between the patients and controls in terms of age (P > 0.05).

The genotype distributions of 1793GG MTHFR (95; 93.25%) and 1793GA (5; 6.75%) were similar in the two groups and were in Hardy-Weinberg equilibrium, as established using the chisquare test (*X ^2^*) (Table 1).

**Table 1:** Genotypic and allelic frequencies of the G1793A

	Control group	Test group
1793GG	95 (n = 38)	93.25 (n = 69)
1793GA	5 (n = 2)	6.75 (n = 5)
1793AA	0.0 (n = 0)	0.0 (n = 0)
G allele (%)	97.5	96.6
A allele (%)	2.5	3.4
P-value (*X*2)	0.03	0.09

### Analysis on the genotypic groups

The genotypic groups 1793GG (n = 107) and 1793GA (n = 7) were compared regarding the presence of risk factors for atherosclerosis (Table 2) and lipid profile (Table 3), and no significant difference was found.

**Table 2:** Distribution of risk factors for atherosclerosis between the genotypic groups 1793GG and 1793GA

Risk factor	1793GG n = 107	Test group	Z*
Hypertension	72 (67.28%)	6 (85.71%)	1.01
Diabetes	19 (17.75%)	2 (28.57%)	0.70
Dyslipidemia	63 (58.87%)	5 (71.42%)	0.65
Smoking	11 (10.28%)	0 (0%)	0.89
Sedentarism	38 (35.51%)	2 (28.57%)	0.37
Family history of myocardial infarction	76 (71.02%)	3 (42.85%)	1.57
Family history of stroke	41 (38.31%)	2 (28.57%)	0.51
Family history of sudden death	46 (42.99%)	3 (42.85%)	0.006

* The tabulated Z value for a risk of error of 5% was 1.96

**Table 3:** Analysis of the concentrations of biochemical parameters in the presence or absence of the G1793A polymorphism

	1793GG n = 107	1793GA n = 7	P*
	Mean	Mean	
Total cholesterol	202 (45)	185 (42)	0.26
High-density lipoprotein	48 (12)	48 (12)	0.92
Triglycerides	158 (89)	162 (53)	0.48
Low-density lipoprotein	124 (39)	110 (34)	0.35
Blood glucose	109 (35)	130 (59)	0.39

* Mann-Whitney test with a significance level of P < 0.05.

### DISCUSSION

Analysis on the molecular data showed that the numerical difference between the allele frequencies found for the A allele of G1793A polymorphism in the groups studied was very small (Table 1). In the test group, the frequency was 3.4%, while in the control, the frequency was 2.5%. The rarity of the 1793A allele explains why both groups were in Hardy-Weinberg equilibrium even in the absence of AA homozygotes. The test of difference between two proportions showed Z = 0.39703467, while the tabulated Z value for a risk of error of 5% was 1.96. It was not possible to affirm that there was increased occurrence of the G1793A mutation among the patients with early CAD, and in this case, the difference between the proportions would have occurred by chance. The frequency of the G1793A mutation in the control group was similar to that found in the Ashkenazi Jewish population,^14^ while in the test group, the frequency was closer to that found among African-Americans^14^ (Table 1). There have been reports in which the 1793A frequency was significantly higher, as found in the mothers of non-syndromic cleft lip and/or palate children (25.9%),^15^ and in end-stage renal disease patients presenting cardiovascular complications (15%).^17^ The frequency of heterozygous G1793A carrier detected in a Chinese population with gastric cancer was 14%, and this figure did not differ from the control group.^19^ These numerical results were very significantly different to the data obtained from the population of the present study, and such differences certainly reflect the influence of ethnic and geographical origin on the transmission of genetic traits.

It is interesting to note that in most studies that have analyzed this polymorphism, it was rare to identify homozygotes. This may partly be explained by the low frequency in most ethnic groups.^14^

A comparison of the risk factors for atherosclerosis present in the patients with normal genotype and heterozygotes showed that there was no significant difference between the proportions (Table 2). This suggests that the population in the G1793A polymorphism analysis did not predispose towards the atherosclerosis risk factors investigated.

Comparison of the means and medians of the parameters of total cholesterol, LDL cholesterol, HDL cholesterol, triglycerides and glucose between normal homozygous and heterozygous groups showed that the presence of the polymorphism did not affect these parameters (Table 3).

The low frequency of the polymorphism complicates the interpretation of Tables 2 and 3, since it was necessary to compare groups with very different numbers of individuals and also using very low total numbers (n = 7), which certainly hampered the statistical analysis. To circumvent this problem, we would need to extend the number of patients, thus increasing the population of heterozygotes in the analysis. 

This study evaluated the frequency of MTHFR G1793A within the context of a limited population, which restricted the number of subjects in the experimental group and prevented the possibility of categorization of information regarding the patients' gender. 

Prevention of CAD is dependent on identifying risk factors. The contribution of molecular biology in this field is crucial, since the genetic markers can be identified before the clinical manifestation occurs, thus making it possible to conduct risk analysis on healthy individuals. The MTHFR gene has been considered as a candidate for contributing towards CAD risk. Casecontrol studies like this may identify targets for future diagnostic and and therapeutic approaches.

Considering the low frequency of the 1793A allele, future research should explore the clinical implication of this polymorphism in larger populations, taking into account the patients' gender, since different clinical features differentiate the male and female risk of CAD.

### CONCLUSIONS

The frequency of the 1793A allele in the test group (3.4%) was similar to that of the control group (2.5%). No significant correlations were found between G1793A polymorphism and occurrences of early coronary events in this population.

## References

[1]  Arruda VR,  von Zuben PM,  Chiaparini LC,  Annichino-Bizzacchi JM,  Costa FF (1997). The mutation Ala677-->Val in the methylene tetrahydrofolate reductase gene: a risk factor for arterial disease and venous thrombosis. Thromb Haemost..

[2]  Gülec S,  Aras O,  Akar E (2001). Methylenetetrahydrofolate reductase gene polymorphism and risk of premature myocardial infarction. Clin Cardiol..

[3]  Kerkeni M,  Addad F,  Chauffert M (2006). Hyperhomocysteinaemia, methylenetetrahydrofolate reductase polymorphism and risk of coronary artery disease. Ann Clin Biochem..

[4]  Alam MA,  Husain SA,  Narang R (2008). Association of polymorphism in the thermolabile 5, 10-methylene tetrahydrofolate reductase gene and hyperhomocysteinemia with coronary artery disease. Mol Cell Biochem..

[5]  Tripathi R,  Tewari S,  Singh PK,  Agarwal S (2010). Association of homocysteine and methylene tetrahydrofolate reductase (MTHFR C677T) gene polymorphism with coronary artery disease (CAD) in the population of North India. Genet Mol Biol..

[6]  Castro R,  Rivera I,  Blom HJ,  Jakobs C,  Tavares de Almeida I (2006). Homocysteine metabolism, hyperhomocystenemia and vascular disease: an overview. J Inherit Metab Dis..

[7]  Wald DS,  Law M,  Morris JK (2002). Homocysteine and cardiovascular disease: evidence on causality from a meta-analysis. BMJ..

[8]  Jakubowski H (2006). Pathophysiological consequences of homocysteine excess. J Nutr..

[9]  Wald DS,  Law M,  Morris JK (2004). The dose-response relation between serum homocysteine and cardiovascular disease: implications for treatment and screening. Eur J Cardiovasc Prev Rehabil..

[10]  Kluijtmans LA,  van den Heuvel LP,  Boers GH (1996). Molecular genetic analysis in mild hyperhomocysteinemia: a common mutation in the methylenetetrahydrofolate reductase gene is a genetic risk factor for cardiovascular disease. Am J Hum Genet..

[11]  Freitas AI,  Mendonça I,  Guerra G (2008). Methylenetetrahydrofolate reductase gene, homocysteine and coronary artery disease: the A1298C polymorphism does matter. Inferences from a case study (Madeira, Portugal). Thromb Res..

[12]  Kölling K,  Ndrepepa G,  Koch W (2004). Methylenetetrahydrofolate reductase gene C677T and A1298C polymorphisms, plasma homocysteine, folate, and vitamin B12 levels and the extent of coronary artery disease. Am J Cardiol..

[13]  Szczeklik A,  Sanak M,  Jankowski M (2001). Mutation A1298C of methylenetetrahydrofolate reductase: risk for early coronary disease not associated with hyperomocysteinemia. Am J Med Genet..

[14]  Rady PL,  Szucs S,  Grady J (2002). Genetic polymorphisms of methylenetetrahydrofolate reductase (MTHFR) and methionine synthase reductase (MTRR) in ethnic populations in Texas: a report of a novel MTHFR polymorphic site, G1798A. Am J Med Genet..

[15]  Bufalino A,  Ribeiro Paranaíba LM,  Nascimento de Aquino S (2010). Maternal polymorphisms in folic acid metabolic genes are associated with nonsyndromic cleft lip and/or palate in the Brazilian population. Birth Defects Res A Clin Mol Teratol..

[16]  Safarinejad MR,  Safarinejad S,  Shafiei N (2010). Role of methylenetetrahydrofolate reductase gene polymorphisms (C677T, A1298C, and G1793A) in the development of early onset vasculogenic erectile dysfunction. Arch Med Res..

[17]  Poduri A,  Mukherjee D,  Sud K (2008). MTHFR A1298C polymorphism is associated with cardiovascular risk in end stage renal disease in North Indians. Mol Cell Biochem..

[18]  Miller SA,  Dykes DD,  Polesky HF (1988). A simple salting out procedure for extracting DNA from human nucleated cells. Nucleic Acids Res..

[19]  Shen H,  Newmann AS,  Hu Z (2005). Methylenetetrahydrofolate reductase polymorphisms/haplotypes and risk of gastric cancer: a case-control analysis in China. Oncol Rep..

